# A QUALITATIVE STUDY EXPLORING TECHNOLOGICAL SUPPORT IN THE DISTANCE CAREGIVING CONTEXT

**DOI:** 10.1093/geroni/igae098.3777

**Published:** 2024-12-31

**Authors:** Andrea Budnick, Farina Bünning

**Affiliations:** Charité - Universitätsmedizin Berlin, Berlin, Berlin, Germany; Charité - Universitätsmedizin Berlin, Berlin, Berlin, Germany

## Abstract

As demographic change progresses, fewer and fewer relatives are available to provide care locally. Consequently, distance caregiving has increasingly attracted the attention of the gerontological research community. Technological support plays a role in enabling the distance caregiving arrangement. However, there is a paucity of research on this type of support in the distance caregiving context. Hence, this qualitative study explores actors’ experiences, perceptions, and attitudes in performing their roles within the distance caregiving arrangement by utilizing technological support. One-on-one guided interviews were conducted face-to-face with distance caregivers (N=20; mean age=51.8 years), care recipients (N=20; mean age=82.4 years), and representatives of the supportive local network (N=18; mean age=55.4 years). Interviews were transcribed verbatim. Data were analyzed using phenomenological analysis. Our findings show that distance caregivers successfully implement various forms of communication technology to keep the care arrangement running by using devices like landline phone, smartphone, and/or e-mail. In everyday life, aids such as dementia clocks, smart home applications for automated door and/or window control, or smart speakers are used. Around-the-clock video surveillance of care recipients is used in only two distance caregiving arrangements. Potentials and risks of technological support are viewed by all actors and include maintaining independence or social participation, but concerns are also expressed regarding the triggering of false alarms, possible misuse, or data protection. Fear of technology or lack of tech literacy prevent actors from using technological support. Triangulated findings imply that improved tech literacy could create more reliability and simplify distance caregiving for all actors involved.

